# Forsythiaside A Regulates Activation of Hepatic Stellate Cells by Inhibiting NOX4-Dependent ROS

**DOI:** 10.1155/2022/9938392

**Published:** 2022-01-05

**Authors:** Mengting Zhou, Xingtao Zhao, Li Liao, Ying Deng, Meichen Liu, Jing Wang, Xinyan Xue, Yunxia Li

**Affiliations:** State Key Laboratory of Southwestern Chinese Medicine Resources; Key Laboratory of Standardization for Chinese Herbal Medicine, Ministry of Education; School of Pharmacy, Chengdu University of Traditional Chinese Medicine, Chengdu 611137, China

## Abstract

Hepatic stellate cells (HSCs) activation is an important step in the process of hepatic fibrosis. NOX4 and reactive oxygen species expressed in HSCs play an important role in liver fibrosis. Forsythiaside A (FA), a phenylethanoid glycoside extracted and isolated from Forsythiae Fructus, has significant antioxidant activities. However, it is not clear whether FA can play a role in inhibiting the HSCs activation through regulating NOX4/ROS pathway. Therefore, our purpose is to explore the effect and mechanism of FA on HSCs activation to alleviate liver fibrosis. LX2 cells were activated by TGF-*β*1 *in vitro*. MTT assay and Wound Healing assay were used to investigate the effect of FA on TGF-*β*1-induced LX2 cell proliferation and migration. Elisa kit was used to measure the expression of MMP-1 and TIMP-1. Western blot and RT-qPCR were used to investigate the expression of fibrosis-related COLI, *α*-SMA, MMP-1 and TIMP-1, and inflammation-related TNF-*α*, IL-6 and IL-1*β*. The hydroxyproline content was characterized using a biochemical kit. The mechanism of FA to inhibit HSCs activation and apoptosis was detected by DCF-DA probe, RT-qPCR, western blot and flow cytometry. NOX4 siRNA was used to futher verify the effect of FA on NOX4/ROS pathway. The results showed that FA inhibited the proliferation and migration of LX2 cells and adjusted the expression of MMP-1, TIMP-1, COLI, *α*-SMA, TNF-*α*, IL-6 and IL-1*β* as well as promoted collagen metabolism to show potential in anti-hepatic fibrosis. Mechanically, FA down-regulated NOX4/ROS signaling pathway to improve oxidation imbalances, and subsequently inhibited PI3K/Akt pathway to suppress proliferation. FA also promoted the apoptosis of LX2 cells by Bax/Bcl2 pathway. Furthermore, the effects of FA on TGF-*β*1-induced increased ROS levels and *α*-SMA and COLI expression were weaken by silencing NOX4. In conclusion, FA had potential in anti-hepatic fibrosis at least in part by remolding of extracellular matrix and improving oxidation imbalances to inhibit the activation of HSCs and promote HSCs apoptosis.

## 1. Introduction

Liver fibrosis was the abnormal proliferation of connective tissue and the excessive accumulation of extracellular matrix proteins in the liver caused by various pathogenic factors, which usually occurred in most types of chronic liver disease such as hepatitis B and C, alcohol liver, nonalcoholic liver disease, cholestasis and autoimmune hepatitis [1, 2]. If left untreated, liver fibrosis might develop into cirrhosis, liver cancer, and liver failure [3]. Its morbidity and mortality rates were increasing year by year. However, to date, there was no approved anti-hepatic fibrosis therapy [4].

Hepatic stellate cells (HSCs) were the main types of fibrotic cells in the liver and a major source of extracellular matrix, such as collagen (COL) I and COL III. Persistent hepatocyte injury and inflammatory responses led to the activation of HSCs which represented a central event in liver fibrosis. In addition, multiple factors directly or indirectly induced HSCs activation, such as fibrotic tissue microenvironment, intestinal malnutrition, immune and systemic metabolic abnormalities and hepatitis virus products and so on [5]. Under stimulation, HSCs transformed from resting-state into a myofibroblast-like phenotype which showed the ability to proliferate and migrate. Therefore, targeted drug molecular intervention to induce HSCs inactivation therapeutically was a more effective and less toxic precise anti-hepatic fibrosis therapy [5].

Endogenous reactive oxygen species (ROS) which were catalyzed by nicotinamide adenine dinucleotide phosphate oxidase (NOX) enzyme complexes played an important role in endothelial-mesenchymal transition and induced the activation of HSCs [6]. The NOX family mainly consisted of 7 subunits, namely phagocytic NOX2 and non-phagocytic NOX1, NOX3, NOX4, NOX5 as well as dual oxidase Duox proteins (Duox1 and Doux2) [7]. Among them, NOX4 was highly expressed in HSCs. More and more research showed that the increase of NOX4 activity and ROS production resulted in oxidative stress to promote HSCs activation and subsequently lead to liver fibrosis [8–12]. And then a series of signal transduction downstream were adjusted such as TGF-*β*/Smads signaling, toll-like receptor signaling, PI3K/Akt pathway [13–15]. Abundant evidence showed that PI3K/AKT signaling cascade played an important role in regulating multiple biological processes, including cell proliferation, metabolism, protein synthesis, autophagy and apoptosis as well as survival, which might be influenced by ROS level [16, 17]. It also affected the synthesis of extracellular matrix in liver, which was closely related to the occurrence and development of liver fibrosis [18, 19]. In addition, it had been shown that the activation of apoptosis (Bcl-2) was also associated with the PI3K/Akt signaling pathway. Therefore, NOX4/ROS pathway may be a good target to regulate activation and apoptosis of HSCs, and thereby alters the life course of activated HSCs.

The rich chemical components and pharmacological effects of traditional Chinese medicine provided an abundant source for the screening of anti-liver fibrosis drugs, with significant efficacy and few side effects. At present, flavonoids, alkaloids, saponins and polysaccharides have shown significant anti-hepatic fibrosis effects by inhibiting hepatitis, suppressing lipid peroxidation, regulating the activation and proliferation of HSCs, promoting HSCs apoptosis and adjusting the degradation of extracellular matrix [20]. Forsythiaside A (FA), an effective component isolated from traditional Chinese medicine *Forsythia suspensa* (Thunb.) Vahl, has shown significant anti-inflammatory, antiviral and antioxidant effects [21–24]. Moreover, it has been shown that FA can protect mice against liver injury induced by LPS/D-Galactosamine by activating Nrf2 and inhibiting NF-*κ*B signaling pathway [25]. However, whether FA can treat liver fibrosis has not been studied so far, and whether it plays an anti-hepatic fibrosis role through NOX4/ROS pathway needs to be researched. Therefore, we used the TGF-*β*1 induced-LX2 cells model to study the effect and possible mechanism of FA on activated HSCs. It provides a preliminary and scientific basis for the development of new safe and effective anti-liver fibrosis drugs.

## 2. Materials and Methods

### 2.1. Reagents and Chemicals

FA (purity above 99%) was obtained from Chengdu MUST Bio-technology Co., Ltd. (Chengdu, China). TGF-*β*1 was purchased from Novoprotein Scientific Inc. (Shanghai, China). LX2 cells were purchased from the Cell Bank of the Xiangya Central Experiment Laboratory of Central South University (Changsha, China) and L02 cells were purchased from Procell Life Science&Technology Co., Ltd. (Wuhan, China). 1640 medium was obtained from Gibco (Australia) and Fetal Bovine Serum was purchased from Zhejiang Tianhang Biological Technology Co. Ltd. BCA Protein Assay Kit, MMP-1 Elisa kits, TIMP-1 Elisa kits and PMSF were obtained from Multi Sciences Biotech Co., Ltd. (Hangzhou, China). Hydroxyproline commercial kit was obtained from Nanjing Jiancheng Bioengineering Institute (Nanjing, China). DCF-DA was obtained from Yeasen Bio-technology Co., Ltd. (Shanghai, China). SiRNA Oligo and GP-siRNA-Mate plus transfection reagent were purchased from Shanghai GenePharma (Shanghai, China). Total RNA Isolation kit, RT Easy™ II and Real Time PCR Easy™-SYBR Green I were purchased from FOREGENE Biotechnology Co. Ltd. (Chengdu, China). Protein phosphatase inhibitor and Protease inhibitor mixture were purchased from Solarbio Sciences & Technology Co., Ltd (Beijing, China). RIPA lysis buffer was obtained from Beyotime Biotechnology (Shanghai, China). Rabbit anti-collagen I, rabbit anti-*α*-SMA, rabbit anti-MMP-1, rabbit anti-TIMP-1, rabbit anti-p-PI3K, rabbit anti-PI3K, rabbit anti-p-Akt, rabbit anti-Akt, rabbit anti-NOX4, rabbit anti-p22^phox^, rabbit anti-Bax, rabbit-anti-Bcl2, rabbit anti-GAPDH, rabbit anti-*β*-actin antibodies and Goat anti-Rabbit IgG-HRP were all obtained from Affinity Biosciences. Annexin-V-FITC Apoptosis Detection Kit was purchased from Beyotime Biotechnology.

### 2.2. Cell Culture and Model Establishment

LX2 cells were cultured in 1640 medium supplemented with 10% fetal bovine serum (FBS) (v/v) and 1% antibiotics. L02 cells were maintained in DMEM medium supplemented with 10% FBS and 1% antibiotics. Cells were maintained in a humidified incubator with 5% CO2 at 37°C and then seeded in 96-well plates and 6-well plates for further experiment. LX2 cells were starved in serum-free 1640 medium for 12 h before the stimulation of appointed concentrations of TGF-*β*1 for 24 h to activate them [26].

### 2.3. Cell Viability Assay

The cell viability assay was performed using the MTT colorimetric assay. Briefly, LX2 or L02 cells were seeded at 5 × 10^3^ cells in each well of 96-well plates. FA was dissolved directly in medium containing 1% FBS and diluted to appointed concentration by double dilution method. Cells at approximately 80% confluence were added with the different concentrations of FA solution and/or TGF-*β*1 (2.5, 5, 10 ng/mL) for 24 h. Then 20 *μ*l MTT solution was added to each well and incubated for an additional 4 hours. Subsequently, the supernatant was carefully sucked away and 150ul DMSO was added to each well to dissolve the crystals. The microplate reader was used to measure the absorbance at 490 nm.

### 2.4. Wound-Healing Assay

LX2 was seeded in six-well plates at 8 × 10^4^ cells in each well in 1640 medium containing 10% FBS. When LX2 cells adhered to the wall as a thin layer, cells were scratched with sterile pipette tips and washed with PBS to remove cell debris. Then LX2 cells were treated with 10 ng/mL TGF-*β*1 in the presence or absence of FA (25, 50, 100 *μ*mol/L) for 24 h and the wound was photographed under an inverted microscope at 0 h, 12 h, 18 h and 24 h, respectively. Cell migration was quantified by measuring the area of the wound and relative migration rate which was calculated by [W (0 h) - W (24 h/18 h/12 h)]/W (0 h) % equation.

### 2.5. ELISA Detection

LX2 was seeded in six-well plates at 8 × 10^4^ cells in each well in 1640 medium containing 10% FBS. Cells at approximately 80% confluence were starved for 12 h and then added with 10 ng/mL TGF-*β*1 in the presence or absence of FA solution (25, 50, 100 *μ*mol/L) for 24 h. Cell culture supernatant was used to detect the content of MMP-1 and TIMP-1 by ELISA kits according to the manufacturer's instructions. The absorption wavelength was 450 nm and the correction wavelength was 630 nm detected by a microplate reader. The experiment was repeated three times independently.

### 2.6. Hydroxyproline Content Analysis

The supernatant was collected after the cells were treated according to the “2.5” section. And then hydroxyproline commercial kit was used to detect the content of hydroxyproline in cell culture supernatant according to the manufacturer's instructions. The absorption wavelength was 550 nm detected by a microplate reader.

### 2.7. Intracellular ROS Analysis

2,7-dichlorodihydrofluorescein diacetate (DCF-DA) was used to detect intracellular ROS production. LX2 was cultured in a six-well plate at 8 × 10^4^ cells in each well in 1640 medium containing 10% FBS. Cells at approximately 80% confluence were starved for 12 h and then treated with 10 ng/mL TGF-*β*1 in the presence or absence of 25, 50 and 100 *μ*mol/L FA for 24 h. The cells were then washed twice with PBS and incubated at 37°C in 5% CO2 with 5 *μ*M DCF-DA for 30 min. Subsequently, cells were rinsed carefully with precooled PBS three times and photographed under an inverted fluorescence microscope. ImageJ software was used to analyze the mean fluorescence intensity.

### 2.8. Transfection of siRNA

LX2 cells were cultured in a six-well plate at 8 × 10^4^ cells and NOX4 siRNA transfection was performed by using GP-transfect-Mate reagent according to the manufacturer's instructions when cells were at approximately 60% confluence. Briefly, RNA oligo and transfection reagent were diluted using serum-free and antibiotic-free 1640 medium. After standing for 5 min, the diluted transfection reagent was added to the diluted RNA oligo to prepare the transfection complex and incubated for 15 min before being applied to transfection. The NOX4 siRNA sequences were as follows: sense 5'-CCAUGUGCCGAACACUCUUTT-3' and antisense 5'-AAGAGUGUUCGGCACAUGGTT-3'. A scrambled RNA duplex was used for negative control. After 24 h transfection, LX2 cells activation and FA treatment were performed as “2.5”, and then total protein was extracted for subsequent western blot analysis.

### 2.9. RT-qPCR Analysis

We used RT-qPCR to detect and quantify the gene expression of *α*-SMA, COLI, MMP-1, TIMP-1, TNF-*α*, IL-6, IL-1*β*, NOX4, p22^phox^, Bax, Bcl2. In brief, LX2 was treated as the “2.5” section. After 24 h administration, cells were discarded culture supernatant and washed twice with PBS, and then total RNA was extracted using the Total RNA Isolation kit according to the manufacturer's instructions. Finally, it was resuspended by 50 *μ*L RNase-free water. The purity of RNA was measured by the OD value of OD260/280 nm using the Nucleic Acid/Protein Analyzer. RT Easy™ II was used for cDNA synthesis at the conditions of 42°C for 15 min and 85°C for 5 min according to the manufacturer's instructions. The RT-qPCR conditions were performed at 95°C for 3 min, 40 cycles of 95°C for 10 s and 65°C for 30 s. Each experiment was conducted with three separate biological samples and the formula of 2^-*ΔΔ*Ct^ was used to calculated relative mRNA expression levels of the target genes. All primer sequences were listed in Table 1.

### 2.10. Western Blot Analysis

LX2 was cultured according to the “2.5” section. After 24 h administration, cells were washed with PBS twice and then added to lysis buffer (RIPA lysis buffer: protein phosphatase inhibitor: PMSF: protein mixing enzyme inhibitor =100: 1 : 1 : 1). The cells were crushed by ultrasonic cell crushing apparatus for 3 min and centrifuged at 13400 g for 10 min in 4°C. Then BCA kit was used to detect the protein concentration and then protein concentration was adjusted to be consistent. Protein loading buffer was added to sample proteins for protein denaturation which persisted for 5 min at 100°C. The sample proteins of equal amounts were loaded to polyacrylamide gel electrophoresis and then transferred to PVDF membrane in the ice bath. Subsequently, the membrane was blocked at room temperature in TBST containing 5% non-fat milk for 2 h and incubated with primary antibodies against *α*-SMA, COLI, MMP-1, TIMP-1, NOX4, p22^phox^, PI3K, p-PI3K, Akt, p-Akt, Bax, Bcl2, GAPDH, *β*-actin at 4°C overnight. The next day, the membrane was cleaned by TBST three times and incubated with secondary antibodies for 1 h at 37°C. The gel imager was used to collect the signal and ImageJ software was used to analyze the net optical density.

### 2.11. Apoptosis Analysis by Flow Cytometry

Cells apoptosis was detected using an Annexin-V FITC kit by flow cytometry according to manufacturer's guidelines. Briefly, 24 h after administration, cells were digested with trypsin and collected by centrifugation. Cells were washed with PBS and then suspended in a binding buffer. Subsequently, cells were stained with Annexin-V and PI for 15 min in a dark place and then analyzed by flow cytometry.

### 2.12. Statistical Analysis

All experimental data were presented by mean ± S.D. and analyzed by statistical software SPSS 25.0. *P*-value<0.05 was regarded as a significant difference and differences among the groups were evaluated statistically using One-Way ANOVA.

## 3. Results

### 3.1. TGF-*β*1 Induced the Proliferation of LX2 Cells and Promoted the Secretion of Fibrosis Cytokines *α*-SMA and COLI

**Figure 1:**
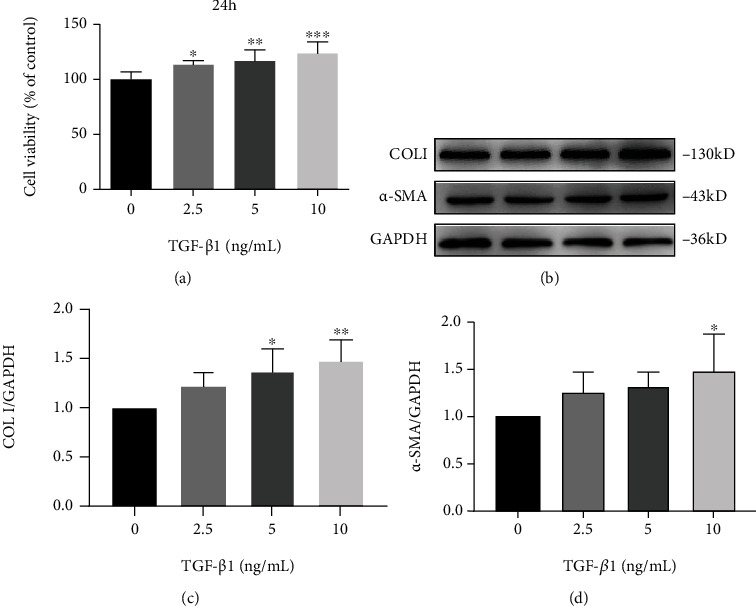
TGF-*β*1 induced LX2 cells proliferation and fibrogenic gene expression. Cells were treated with TGF-*β*1 at 0-10 ng/mL for 24 h. (a) Effect of TGF-*β*1 on cell proliferation was measured by MTT. (B-D) The protein expression of COL I and *α*-SMA was analyzed by western blot assay. Data from the three independent trials were represented by mean ± S.D. ∗∗∗*p* <0.001 TGF-*β*1 vs control; ∗∗*p* <0.01 TGF-*β*1 vs control; ∗*p* <0.05 TGF-*β*1 vs control.

To determine the optimal modeling concentration, 2.5, 5, 10 ng/mL TGF- *β*1 was used to stimulate LX2 cells. As shown in Figure 1(a), different concentrations of TGF-*β*1 all promoted the proliferation of LX2 cells, with the maximum proliferation of 10 ng/mL, which indicated the occurrence of HSCs activation [27]. The expression of the fibrosis-related factors COLI and *α*-SMA in LX2 cells was further measured by western blot assay. As shown in Figures 1(b)–1(d), TGF-*β*1 significantly induced the expression of COLI and *α*-SMA in a dose-dependent manner. However, there was no statistical significance of increased *α*-SMA expression in 2.5 ng/mL and 5 ng/mL groups. Therefore, 10 ng/mL TGF-*β*1 was selected as the optimal modeling concentration to induce HSCs activation.

### 3.2. FA Inhibited the Proliferation and Migration of LX2 Cells

**Figure 2:**
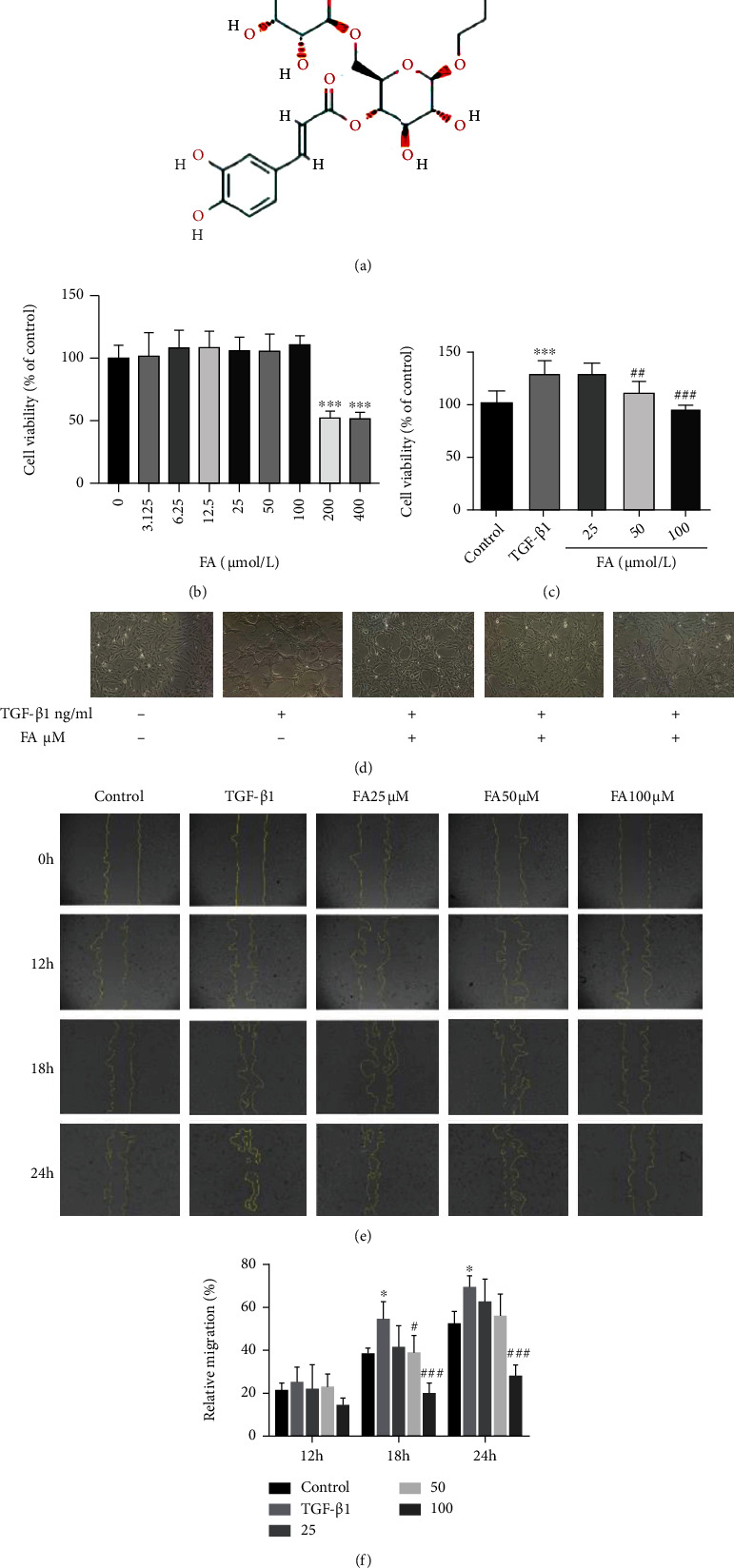
FA inhibited the proliferation and migration of LX2 cells. (A) The chemical formula of FA (https://pubchem.ncbi.nlm.nih.gov/). (B) Effects of different concentrations of FA (0-400 *μ*mol/L) on LX2 cell viability. ∗∗∗*p* <0.001 FA vs control (C) The effects of FA and TGF-*β*1 treatment on cell viability. (D) The morphology of LX2 cells was observed under an inverted microscope (100X). (E) Cell migration was observed at 0 h, 12 h, 18 h, 24 h after FA treatment (100X). Data from the four independent trials. (F) Relative migration rate was used as a quantitative measure of cell migration. Data were represented by mean ± S.D. ∗∗∗*p* <0.001 TGF-*β*1 vs control; ∗*p* <0.05 TGF-*β*1 vs control. ^###^*p* <0.001 TGF-*β*1 + FA vs TGF-*β*1;^##^*p* <0.01 TGF-*β*1 + FA vs TGF-*β*1; ^#^*p* <0.05 TGF-*β*1 + FA vs TGF-*β*1.

To determine the appropriate concentration of FA which chemical formula was shown in Figure 2(a), an MTT assay was used to detect the influence of 0-400 *μ*mol/L FA on cell survival rate. When the concentration was 200 *μ*mol/L, cell survival rate significantly decreased. However, when 100 *μ*mol/L or less, there was no effect on cell survival rate (Figure 2(b)), so FA of 25, 50 and 100 *μ*mol/L was selected for follow-up study. The effect of FA on TGF-*β*1-induced LX2 cell proliferation was further measured. As shown in Figure 2(c), the TGF-*β*1-induced cell proliferation was significantly inhibited after 50,100 *μ*mol/L FA were administered. We observed the morphology of LX2 in different groups starved for 24 hours before treatment shown in Figure 2(d). LX2 cells showed an inactive state in the absence of TGF-*β*1 stimulation. After 10 ng/mL TGF-*β*1 was added to the cells, LX2 started to morphological changes accompanied by structural recombination, including gathering, clumping, and stretching and appearing of many cell-free areas. Interestingly, after FA administration, there was a significant recovery in cell morphology and a reduction in cell-free areas. In addition, the migration of LX2 cells was photographed in 0 h, 12 h, 18 h and 24 h after scratching the cells with sterile pipette tips. The area of the wound was used to calculate the relative migration rate. The results showed that TGF-*β*1 increased the migration of LX2 cells which indicated HSCs activation, while FA inhibited the migration process in a dose-dependent manner at 18 h and 24 h (Figures 2(e) and 2(f)). Therefore, FA inhibited the activation of LX2 by suppressing proliferation and migration.

### 3.3. FA Inhibited TGF-*β*1-Induced the Increased Expressions of Fibrosis Cytokines *α*-SMA and COLI

**Figure 3:**
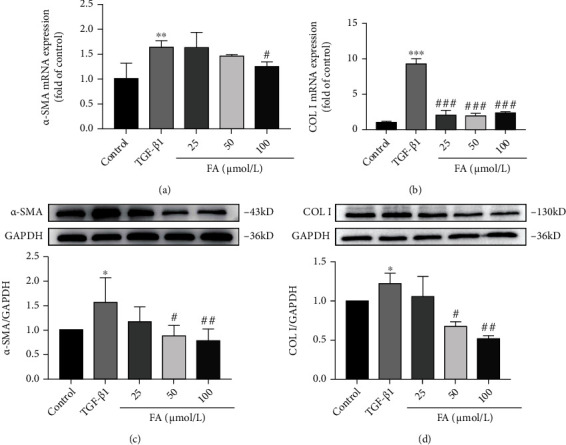
FA inhibited the expression of fibrosis cytokines *α*-SMA and COLI in gene and protein levels. (A-B) The expression of *α*-SMA and COLI mRNA. (C-D) The expression of *α*-SMA and COLI proteins. Data of three independent experiments were represented by mean ± S.D. ∗∗∗*p* <0.001 TGF-*β*1 vs control; ∗∗*p* <0.01 TGF-*β*1 vs control; ∗*p* <0.05 TGF-*β*1 vs control. ^###^*p* <0.001 TGF-*β*1 + FA vs TGF-*β*1; ^##^*p* <0.01 TGF-*β*1 + FA vs TGF-*β*1; ^#^*p* <0.05 TGF-*β*1 + FA vs TGF-*β*1.

To investigate the effect of FA on fibrosis cytokines, we tested the gene and protein expressions of *α*-SMA and COLI after FA treatment for 24 h. The results showed that FA significantly down-regulated the increased expression of *α*-SMA and COLI genes induced by TGF-*β*1 as shown in Figures 3(a) and 3(b). Consistent with RT-qPCR results, FA down-regulated the increased expression of *α*-SMA and COLI in protein levels induced by TGF-*β*1 in a dose-dependent manner (Figures 3(c) and 3(d)). Therefore, FA inhibited the expression of fibrosis cytokines in activated HSCs.

### 3.4. FA Increased Collagen Metabolism in HSCs

**Figure 4:**
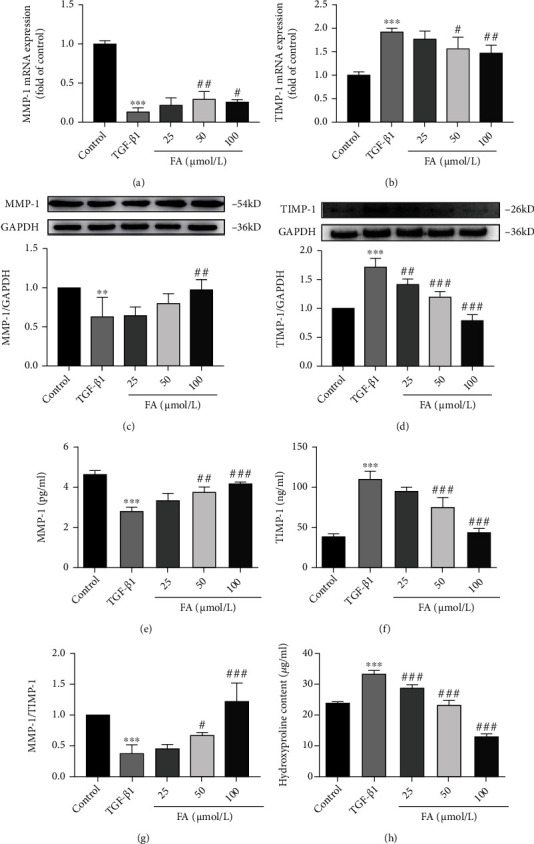
FA promoted collagen metabolism. (A-B) The expression of MMP-1 and TIMP-1 mRNA. (C-D) The expression of MMP-1 and TIMP-1 proteins. (E-G) The content of MMP-1 and TIMP-1 in medium supernatant. (H) The content of hydroxyproline. Data of three independent experiments were represented by mean ± S.D. ∗∗∗*p* <0.001 TGF-*β*1 vs control; ∗∗*p* <0.01 TGF-*β*1 vs control. ^###^*p* <0.001 TGF-*β*1 + FA vs TGF-*β*1;^##^*p* <0.01 TGF-*β*1 + FA vs TGF-*β*1; ^#^*p* <0.05 TGF-*β*1 + FA vs TGF-*β*1.

Matrix metalloproteinases (MMPs) and tissue inhibitors of metalloproteinases (TIMPs) are involved in extracellular matrix metabolism and maintaining the stability of hepatocyte microenvironment together. We measured the expression of MMP-1 and its inhibitor TIMP-1 from different aspects. RT-qPCR analysis results showed that FA significantly up-regulated the expression of MMP-1, while the expression of TIMP-1 was down-regulated in gene levels (Figures 4(a) and 4(b)). Western blot assay was further carried out. It was found that the expressions of MMP-1 and TIMP-1 in protein levels were at the same levels as their genes (Figures 4(c) and 4(d)). In addition, Elisa results further demonstrated that FA reversed TGF-*β*1-induced changes in MMP-1 and TIMP-1. FA increased the TGF-*β*1-induced the down-regulated expression of MMP-1(Figure 4(e)) and decreased the TGF-*β*1-induced the up-regulated expression of TIMP-1(Figure 4(f)). In addition, the MMP-1/TIMP-1 ratio was increased by FA treatment shown in Figure 4(g). All the results indicated that FA modulated MMP-1 and TIMP-1 to reshape extracellular matrix. To investigate the collagen metabolism directly, we measured the content of hydroxyproline in medium supernatant, it was found that FA significantly decreased the content of hydroxyproline compared with TGF-*β*1 group, indicating that FA promoted collagen metabolism process (Figure 4(h)).

### 3.5. FA Decreased the ROS Generation Stimulated by TGF-*β*1

**Figure 5:**
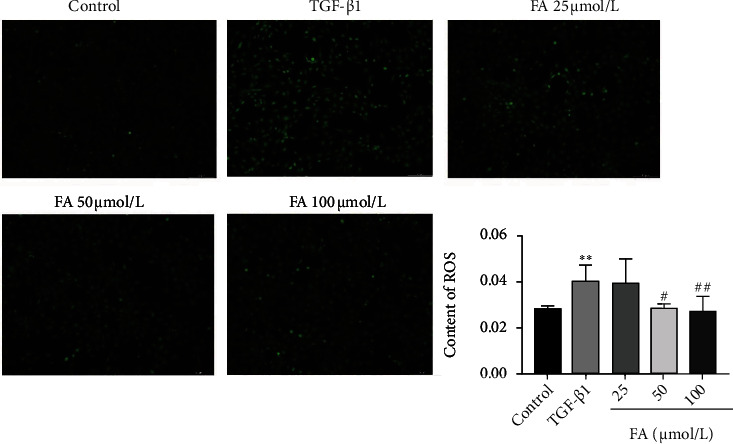
FA decreased the production of ROS in LX2 cells which was detected by an inverted fluorescence microscope (100X). Data from the six independent trials were represented by mean ± S.D. and quantified by ImageJ software. ∗∗*p* <0.01 TGF-*β*1 vs control.^##^*p* <0.01 TGF-*β*1 + FA vs TGF-*β*1; ^#^*p* <0.05 TGF-*β*1 + FA vs TGF-*β*1.

Research has shown that ROS generation is closely related to the development of liver fibrosis [28]. We detected the ROS generation by an inverted fluorescence microscope. As shown in Figure 5, the intensity of green fluorescence represents the ROS content. Compared with control group, TGF-*β*1 induced a significant increase in ROS generation, while FA reversed the process. The mean fluorescence intensity was used to characterize ROS content, which indicated that FA down-regulated the generation of ROS in a dose-dependent manner and improved oxidation imbalances in activated HSCs.

### 3.6. FA Suppressed the Expressions of TNF-*α*, IL-6, IL-1*β*

**Figure 6:**
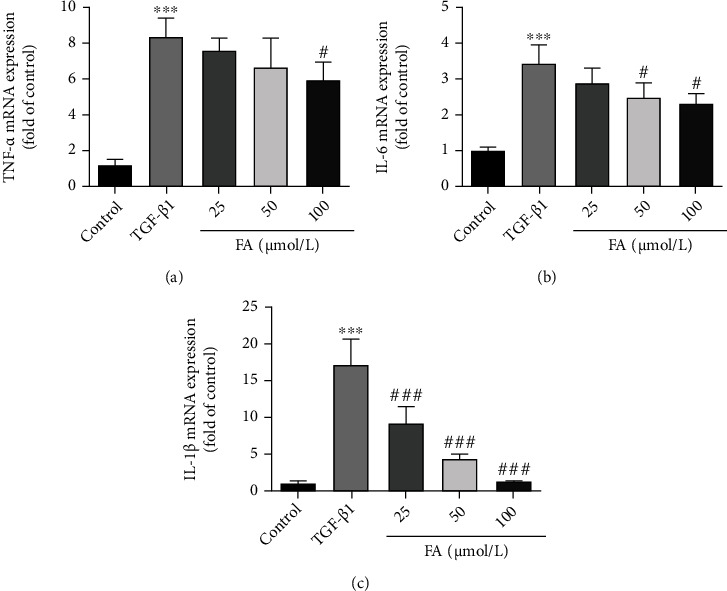
FA inhibited the mRNA expressions of TNF-*α*, IL-6, and IL-1*β*. (A) TNF-*α*; (B) IL-6; (C) IL-1*β*. Data of three independent experiments were represented by mean ± S.D. ∗∗∗*p* <0.001 TGF-*β*1 vs control. ^###^*p* <0.001 TGF-*β*1 + FA vs TGF-*β*1;^#^*p* <0.05 TGF-*β*1 + FA vs TGF-*β*1.

Too much ROS production was involved in the progression of inflammatory disorders [29]. Therefore, we further measured the expression of genes associated with inflammation. As shown in Figure 6, TNF-*α*, IL-6, and IL-1*β* displayed noticeable changes by TGF-*β*1 stimulation, while FA reversed the increased expression of TNF-*α*, IL-6 and IL-1*β* in a dose-dependent manner. All the results indicated that FA attenuated the TGF-*β*1-induced inflammatory disorders.

### 3.7. FA Inhibited the Activation of HSCs through NOX4/ROS Pathway

**Figure 7:**
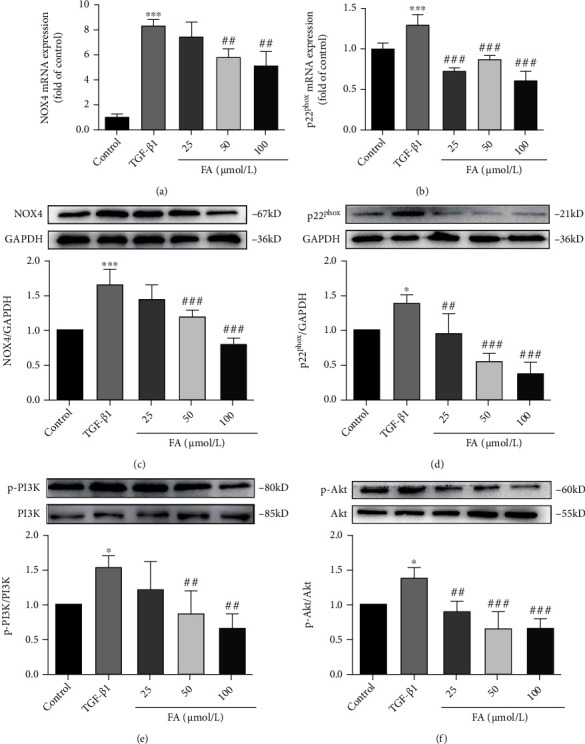
FA inhibited the TGF-*β*1-induced activation of NOX4/ROS and PI3K/Akt pathway. (A-B) The expression of NOX4 and p22^phox^ mRNA. (C-D) The expression of NOX4 and p22^phox^ proteins. (E-F) The phosphorylation levels of PI3K and Akt. Data of three independent experiments were represented by mean ± S.D. ∗∗∗*p* <0.001 TGF-*β*1 vs control; ∗*p* <0.05 TGF-*β*1 vs control. ^###^*p* <0.001 TGF-*β*1 + FA vs TGF-*β*1;^##^*p* <0.01 TGF-*β*1 + FA vs TGF-*β*1.

To investigate the expression of NOX4 and p22^phox^, we conducted RT-qPCR analysis and western bolt assay. It was found that NOX4 and p22^phox^ were significantly increased by TGF-*β*1 stimulation. After FA treatment, NOX4 and p22^phox^ were down-regulated in both gene and protein levels (Figures 7(a)–7(d)).

### 3.8. FA Inhibited the PI3K/Akt Pathway on LX2 to Suppress HSCs Proliferation

Previous studies showed that ROS mediated the activation of PI3K/Akt pathway which was associated to regulate the proliferation, survival, metabolism of cells. Therefore, we further investigated the expression of PI3K, p-PI3K, Akt, p-Akt by western bolt assay. As shown in Figures 7(e)–7(f), FA suppressed the expression of p-PI3K and p-Akt compared with TGF-*β*1 group. Hence, FA could down-regulate the phosphorylation of PI3K and Akt to inhibit the proliferation of LX2.

### 3.9. FA Promoted LX2 Cells Apoptosis through Bax/Bcl2 Pathway

**Figure 8:**
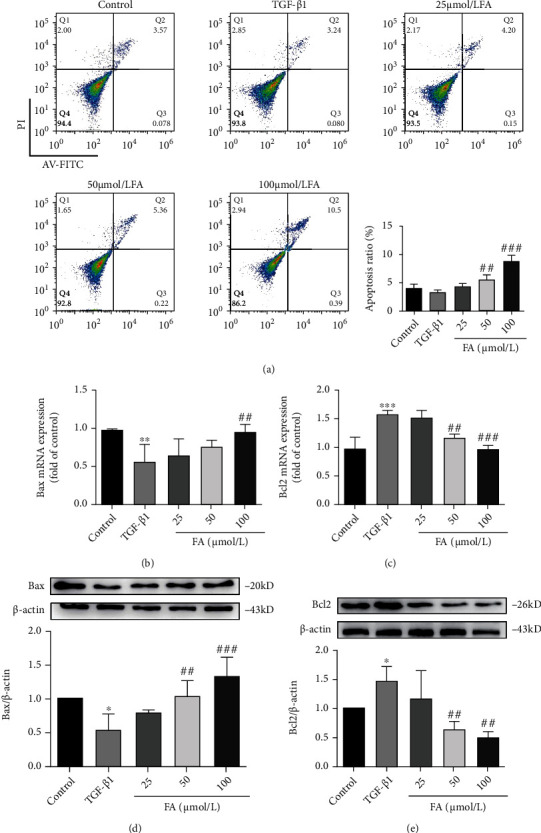
FA promoted the apoptosis of LX2 cells by regulating Bax/Bcl2 pathway. (A) LX2 cells apoptosis was detected by flow cytometry. (B-C) The expression of Bax and Bcl2 mRNA. (D-E) The expression of Bax and Bcl2 proteins. Data of three independent experiments were represented by mean ± S.D. ∗∗∗*p* <0.001 TGF-*β*1 vs control; ∗∗*p* <0.01 TGF-*β*1 vs control; ∗*p* <0.05 TGF-*β*1 vs control. ^###^*p* <0.001 TGF-*β*1 + FA vs TGF-*β*1;^##^*p* <0.01 TGF-*β*1 + FA vs TGF-*β*1.

To investigate the effect of FA on LX2 cells apoptosis, we performed cells apoptosis detection by flow cytometry and the results was shown in Figure 8(a). Q2 and Q3 indicated late and early apoptotic cells, respectively, and their combination indicated the proportion of apoptotic cells. We found that FA promoted LX2 cells apoptosis in a dose-dependent manner. We also detected the expression of Bax and Bcl2 by RT-qPCR and western blot. As shown in Figures 8(b)–8(c), FA significantly down-regulated the mRNA expression of Bcl2 and up-regulated the mRNA expressions of Bax. In addition, western blot assay also proved that FA reversed the decreased expressions of Bax and increased expression of Bcl2 by TGF-*β*1 stimulation, which indicated that FA promoted the apoptosis of HSCs (Figures 8(d)–8(e)).

### 3.10. NOX4 Silence in HSCs Decreased the Effect of FA on ROS Production and Collagen Levels

**Figure 9:**
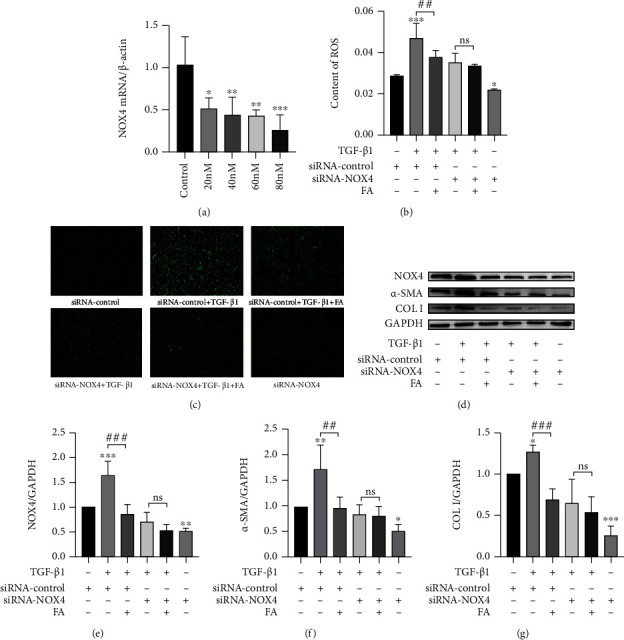
FA inhibited ROS production and HSCs activation partly via inhibiting NOX4 over-expression. (A) SiRNA concentration screening. ∗∗∗*p* <0.001 siRNA-NOX4 group vs control; ∗∗*p* <0.01 siRNA-NOX4 group vs control; ∗*p* <0.05 siRNA-NOX4 group vs control. (B-C) ROS levels measured by an inverted fluorescence microscope (100X) and quantified by ImageJ software. (D-G) The proteins expression of NOX4, *α*-SMA and COL I. ∗∗∗*p* <0.001 vs siRNA-control; ∗∗*p* <0.01 vs siRNA-control; ∗*p* <0.05 vs siRNA-control.^###^*p* <0.001 vs siRNA-control + TGF-*β*1;^##^*p* <0.01 vs siRNA-control + TGF-*β*1.

In order to investigate whether the inhibition of HSCs activation by FA was related to NOX4. We transfected NOX4 siRNA to interfere the expression of NOX4 mRNA, so the expression of NOX4 mRNA was downregulated. First, we detected the optimal dose of NOX4 siRNA. As shown in Figure 9(a), when the concentration reached to 80 nM, NOX4 mRNA expression was inhibited to more than 75%. Therefore, the concentration of 80 nM was selected for NOX4 mRNA silencing. We detected the ROS production by using DCF-DA probe and we found that FA decreased TGF-*β*1-induced ROS production in 1.24 fold in scrambled siRNA groups. However, the decreased ROS production by FA was abolished by NOX4 siRNA (Figures 9(b)–9(c)). It indicated that FA reduced TGF-*β*1-induced increased ROS production partly by inhibiting NOX4 expression.

Then the expression of *α*-SMA and COLI were detected by western blot analysis. In scrambled siRNA groups, the expression of *α*-SMA and COLI were decreased in 1.79 fold and 1.86 fold in FA-treated group compared to TGF-*β*1 group, respectively. However, in NOX4 silencing group, the expression of *α*-SMA and COLI were decreased only in 1.06 fold and 1.18 fold in FA-treated group compared to TGF-*β*1 group, respectively (Figures 9(d)–9(g)). All the results indicated that FA deprived TGF-*β*1-induced HSCs activation partly by suppressing NOX4 over-expression.

### 3.11. FA Did Not Affect the Viability of Normal Liver Cells

**Figure 10:**
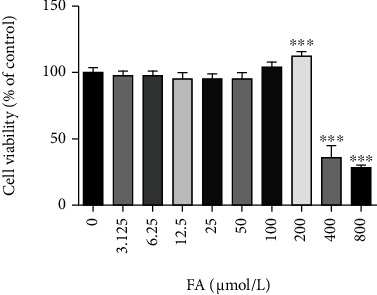
Effects of different concentrations of FA (0-800 *μ*mol/L) on L02 cell viability. Data from the six independent trials were represented by mean ± S.D. ∗∗∗*p* <0.001 FA vs control.

To evaluate the safety of FA in the treatment of liver fibrosis, we used MTT colorimetric assay to detect the effect of FA on the viability of normal liver cells L02. As shown in Figure 10, when the concentration of FA was less than 200 *μ*mol/L, FA had no significant inhibitory effect on the growth of L02 cells. Interestingly, the concentration of 200umol/L promoted the growth of L02 cells. However, 200umol/L FA significantly inhibited the growth of LX2 cells, indicating that FA had no effect on the viability of normal liver cells at no more than 100 *μ*mol/L and it even had the protective effect of promoting the proliferation of normal liver cells at 200 *μ*mol/L.

## 4. Discussion

At present, there were no approved drugs to treat symptoms of liver fibrosis, and studies showed that HSCs-derived cartilage oligomeric matrix proteins were directly involved in the development of hepatocellular carcinoma in coordination with CD36 [30]. Therefore, timely and effective measures might be taken to prevent the further development of liver fibrosis. FA was a phenylethanoid compound isolated from the dried fruit of *Forsythia suspensa* (Thunb.) Vahl. Studies have shown that FA alleviates lipopolysaccharide/d-galactosamine-induced acute liver injury [25] and acetaminophen-induced zebrafish liver injury [31]. All of these indicated that FA had shown significant anti-inflammatory and antioxidant activity to protect liver. However, liver disease is a progressive process from liver injury, hepatitis, fatty liver to fibrosis, cirrhosis, and even liver cancer [32]. The severity and pathogenesis of different stages of liver disease are different. Hence, whether FA can alleviate liver fibrosis is poorly unknown at present. TGF-*β*1, as a major profibrogenic cytokine, induced extracellular matrix expression [33]. In our study, we established TGF-*β*1-induced LX2 cell models which had become a new tool for liver fibrosis analysis [34] *in vitro* to explore the effect of FA on HSCs activation. We first detected the optimal modeling concentration of TGF-*β*1 characterized through cell proliferation and expressions of pro-fibrogenic associated factors. It was found that HSCs were successfully activated by 10 ng/mL TGF-*β*1 stimulation for 24 h *in vitro*. As we all know, activated HSCs are the major cellular source of matrix protein-secreting myofibroblasts which are the major driver of liver fibrogenesis [5]. When TGF-*β*1 induced HSCs activation, the proliferation and migration ability were significantly upregulated, and cell apoptosis was decreased [35]. Our results showed that FA at a safe dose could significantly inhibit the proliferation and migration of LX2 cells induced by TGF-*β*1, indicating that FA could effectively inhibit the activation of HSCs. The activated HSCs transformed into myofibroblasts, morphologically altered, massively synthesized extracellular matrix and expressed *α*-SMA. Collagen was an important component of extracellular matrix. The content of collagen represented the progression and regression of liver fibrosis [36]. Our results showed that FA significantly down-regulated the expression of COLI, hydroxyproline and *α*-SMA. MMPs were a family of enzymes specializing in the degradation of both collagenous and non-collagenous substrates [37]. MMPs were regulated by TIMPs and the MMPs/TIMPs ratio often indicated the degree of extracellular matrix protein degradation and hepatic fibrosis progression [38]. In our study, FA up-regulated the expression of MMP-1 and down-regulated TIMP-1 in both gene and protein levels to alleviate the extracellular matrix deposition.

Excessive ROS production is an important stimulant in the activation of HSCs [39]. ROS was mainly catalyzed by NOX enzyme complexes and NOX4 was abundantly expressed in HSCs. Numerous studies have shown that NOX4-mediated oxidative stress is closely related the liver fibrosis [40]. P22^phox^ formed a complex with ectopic expression of NOX4, which increased the stability of NOX4 and in turn increased the expression of p22^phox^. Accumulating evidence showed that NOX4 was the vital mediator in the activation of HSCs and the development of hepatic fibrosis. In bile duct ligation-induced liver fibrosis, pharmacological NOX4 inhibitor alleviated liver fibrosis, hepatocyte apoptosis and ROS production [40]. Another study suggested that NOX4 expression was increased in TGF-*β*-treated HSCs and CCl_4_-induced mice liver fibrosis. And the expression level of NOX4 was correlated with the degree of liver fibrosis derived from hepatitis C virus [41]. SiRNA interference with NOX4 expression attenuated HSCs activation and NOX4 knockout reversed fibrosis phenotype in activated HSCs. More importantly, knocking down NOX4 decreased the expression *α*-SMA and collagen production in activated HSCs [42]. Phagocytic NOX core enzymes consisted of several different subunits to form active enzyme complexes, including catalytic subunits (gp91^phox^, p40^phox^) to form heterodimeric subunit flavocytochrome b558 in the membrane and regulatory subunit (p47^phox^, p67^phox^, p22^phox^) to form cytoplasmic complexes [43]. When NOX was stimulated by various factors, the subunit p47^phox^ was phosphorylated. Subsequently, cytoplasmic regulatory subunit shifted to cell membrane and interacted with the flavocytochrome b558 complex, resulting in the activation of NOX. In addition, activation of NOX also required the involvement of Rac2 and Rap1A [42, 44]. Finally, the activated NOX used NADPH to transfer electrons to oxygen molecules, leading to the production of ROS. The structure of non-phagocytic NOX (NOX1, NOX3, NOX4, NOX5, DUOX1, and DUOX2) was similar to that of phagocytic NOX2. However, activation of NOX4 required only the recruitment of cytoplasmic regulatory subunit p22^phox^ [45]. Therefore, the discovery of NOX inhibitors that directly target the ROS production pathway may serve as an effective molecular pathway to inhibit HSCs activation [46]. What is noteworthy is that NOX4 isoform plays a vital role in tissue repair functions of myofibroblasts and fibrogenesis, and NOX4-dependent ROS is required for TGF-*β*1-induced myofibroblast differentiation and extracellular matrix production [47]. Therefore, in our study, we investigated the expressions of NOX4 and p22^phox^ in TGF-*β*1-induced LX2 cells and detected the levels of intracellular ROS. We were pleasantly surprised to find that FA could dose-dependently down-regulate the expression of NOX4 and p22^phox^, resulting in the decreased production of ROS. For further verified the key role of the NOX4/ROS pathway, we interfered NOX4 expression with siRNA and found that inhibition of NOX4 expression weakened the effect of FA on ROS production, COLI and *α*-SMA expressions. All the results indicated that FA was a good free radical scavenger by inhibiting NOX4/ROS to inhibit HSCs activation.

NOXs/ROS plays an important role in the pathogenesis of liver fibrosis and is also involved in the regulation of several fibrosis-related pathways, including TGF-*β*/Smad pathway, MAPK pathway, PI3K/Akt pathway, NF-*κ*B pathway and so on [48]. In our study, we found FA inhibited the phosphorylation of PI3K and Akt to suppress the proliferation and migration of LX2 cells. Furthermore, Bax/Bcl2 pathway, the downstream signal transduction pathway of PI3K/Akt pathway, was also regulated by FA to induce apoptosis of HSCs. However, whether the activation of LX2 cells is possible through the synergistic effect of multiple pathways rather than just the PI3K/Akt pathway remains to be further studied.

In addition, our study is only a preliminary *in vitro* experiment to verify the effect of FA on the activation and apoptosis of HSCs. But we found that FA inhibited TGF-*β*1-induced the activation of HSCs by inhibiting NOX4/ROS to improve oxidation imbalances, and then inhibiting PI3K/Akt signaling pathways to suppress HSCs proliferation and Bax/Bcl2 pathway to promote apoptosis for the first time. All the results indicated that FA might have the potential in the treatment of liver fibrosis. Therefore, our research is innovative and of scientific value to some extent. In addition, our study provides reference and inspiration for the in-depth exploration of FA on anti-liver fibrosis. In subsequent studies, we can establish a CCl_4_-induced mice/rats liver fibrosis model *in vivo* for verification. And if other pathways such as Nrf2-ARE [49] are also involved in the treatment of liver fibrosis by FA is still unknown. So we can analyze the differential proteins via high-throughput proteomics to Gene Ontology analysis which is one of the most promising technologies to screen disease biomarkers and mechanism of drug action [50], and then enrich the possible pathways affected by FA through KEGG pathway database, REACTOME pathway database and Wiki pathway database. Therefore, our study provides scientific support and guidelines for future studies on FA against liver fibrosis.

## 5. Conclusion

**Figure 11:**
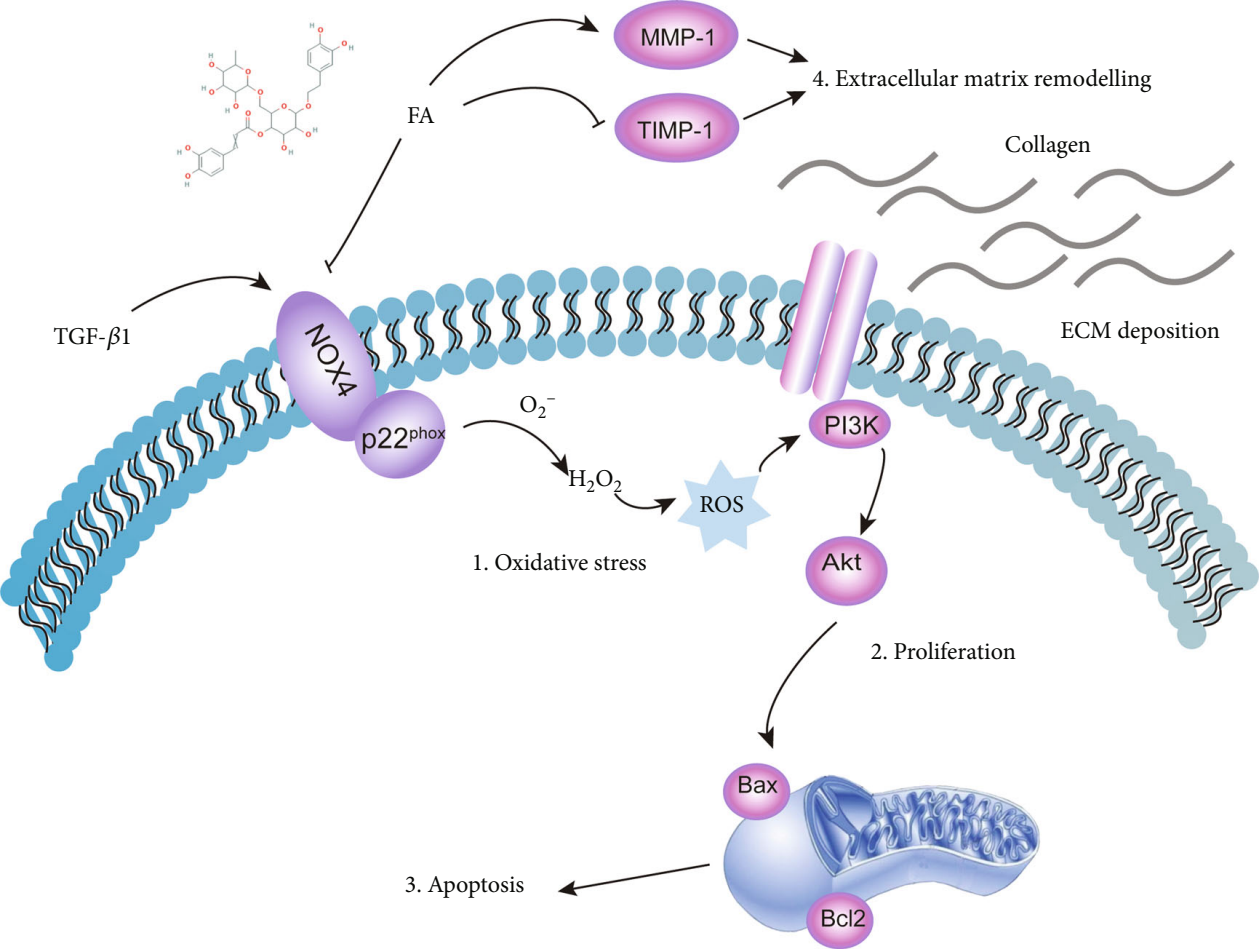
The mechanism of FA on alleviating HSCs activation. FA alleviated TGF-*β*1-induced LX2 cells activation at least in part through four ways. (1) Inhibiting oxidative stress by NOX4/ROS pathway. (2) Modulating proliferation and migration of HSCs by PI3K/Akt pathway. (3) Promoting apoptosis of HSCs by Bax/Bcl2 pathway. (4) Regulating the remolding of extracellular matrix.

Our research demonstrated that FA alleviated TGF-*β*1-induced LX2 cells activation at least in part through remolding of extracellular matrix and improving oxidation imbalances by NOX4/ROS pathway to inhibit the proliferation, migration and promote apoptosis of HSCs (Figure 11). Interference with NOX4 mRNA expression may reverse the progression of HSCs activation. All these results indicated that FA had a potential anti-liver fibrosis efficacy, which provided scientific evidence for future studies on FA against liver fibrosis.

## Figures and Tables

**Table 1:** The gene primer sequence used for RT-qPCR.

Gene	Forward (5´-3´)	Reverse (5´-3´)
*α*-SMA	ACTGCCTTGGTGTGTGACAA	CACCATCACCCCCTGATGTC
COLI	CCTGGATGCCATCAAAGTCT	CGCCATACTCGAACTGGAAT
TNF-*α*	CACAGTGAAGTGCTGGCAAC	GATCAAAGCTGTAGGCCCCA
IL-6	ACAGGGAGAGGGAGCGATA	CCAGTCCTCTTTGTTGGGGAT
IL-1*β*	TGATGGCTTATTACAGTGGCA	CGGAGATTCGTAGCTGGATG
NOX4	CCAGATGTTGGGGGATTGTGT	GAGTGTTCGGCACATGGGTA
p22^phox^	CATCTACCTACTGGCGGCTG	CTTGATGGTGCCTCCGATCT
MMP-1	AGAGCAGATGTGGACCATGC	TTGTCCCGATGATCTCCCCT
TIMP-1	TTTTGTGGCTCCCTGGAACA	AAACAGGGAAACACTGTGCAT
Bax	TGAGCAGATCATGAAGACAGGG	TGAGACACTCGCTCAGCTTC
Bcl2	TCACTTGTGGCCCAGATAGG	GATAACGGAGGCTGGGATGC
*β*-Actin	CTTCGCGGGCGACGAT	CCACATAGGAATCCTTCTGACC
GAPDH	ACTAGGCGCTCACTGTTCT	CCAATACGACCAAATCCGTTG

## Data Availability

The datasets in this study are available to provide by corresponding authors on reasonable request.

## Abbreviations

FA: Forsythiaside A
TGF-*β*1: Transforming growth factor-*β*1
*α*-SMA: *α*-Smooth muscle actin
COLI: Collagen I
HSCs: Hepatic stellate cells
MMP-1: Matrix metalloproteinase-1
TIMP-1: Tissue inhibitor of metalloproteinase 1
TNF-*α*: Tumor necrosis factor-*α*
IL-6: Interleukin-6
IL-1*β*: Interleukin-1*β*
ROS: Reactive oxygen species
NOX4: Nicotinamide adenine dinucleotide phosphate oxidase 4
PI3K: Phosphatidylinositol-3-kinase
Akt: Protein kinase B
Bax: Bcl2 associated X
Bcl2: B-cell lymphoma 2
FBS: Fetal bovine serum
DCF-DA: 2,7-dichlorodihydrofluorescein diacetate.
